# Doping nanoparticles using pulsed laser ablation in a liquid containing the doping agent†

**DOI:** 10.1039/c9na00223e

**Published:** 2019-08-30

**Authors:** Arsène Chemin, Julien Lam, Gaétan Laurens, Florian Trichard, Vincent Motto-Ros, Gilles Ledoux, Vítězslav Jarý, Valentyn Laguta, Martin Nikl, Christophe Dujardin, David Amans

**Affiliations:** Univ Lyon, Univ Claude Bernard Lyon 1, CNRS, Institut Lumière Matière F-69622 Villeurbanne France david.amans@univ-lyon1.fr; Center for Nonlinear Phenomena and Complex Systems, Université Libre de Bruxelles Code Postal 231, Boulevard du Triomphe 1050 Brussels Belgium; Inst Phys AS CR Cukrovarnicka 10 Prague 16200 Czech Republic

## Abstract

While doping of semiconductors or oxides is crucial for numerous technological applications, its control remains difficult especially when the material is reduced down to the nanometric scale. In this paper, we show that pulsed laser ablation of an undoped solid target in an aqueous solution containing activator ions offers a new way to synthesise doped-nanoparticles. The doping efficiency is evaluated for laser ablation of an undoped Gd_2_O_3_ target in aqueous solutions of EuCl_3_ with molar concentration from 10^−5^ mol L^−1^ to 10^−3^ mol L^−1^. Thanks to luminescence experiments, we show that the europium ions penetrate the core of the synthesised monoclinic Gd_2_O_3_ nanoparticles. We also show that the concentration of the activators in the nanoparticles is proportional to the initial concentration in europium ions in the aqueous solution, and a doping of about 1% ([Eu]/[Gd] atomic ratio) is reached. On the one hand, this work could open new ways for the synthesis of doped nanomaterials. On the other hand, it also raises the question of undesired penetration of impurities in laser-generated nanoparticles in liquids.

## Abstract 2

While doping is crucial for numerous technological applications, its control remains difficult especially when the material is reduced down to the nanometric scale. We suggest a new way to dope nanoparticles using laser ablation in liquids.

## Graphical abstract

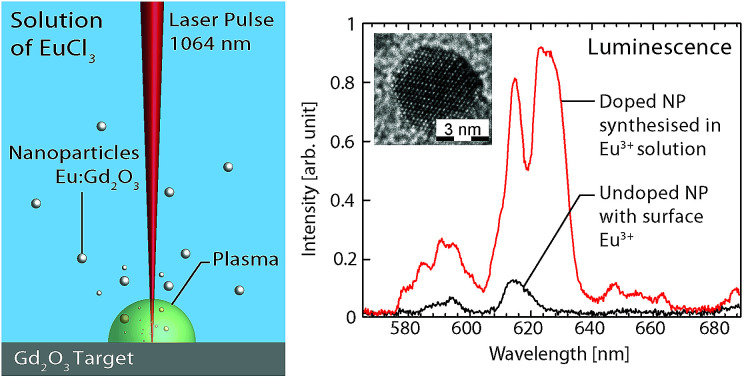

## 1 Introduction

Doping of semi-conductors and oxides has been pivotal in the development of modern technologies as it enables one to finely tune electronic and optical properties of the materials.^1–6^ At the nanometric scale, a crucial glass ceiling remains in the ability to control the doping mechanisms.^7,8^ Indeed, being able to dope on demand nanoparticles with the desired impurities is still challenging because of self-purification mechanisms^9^ or affinity issues between the impurities and the particles' surface during their growth.^10^ In this context, the impurity positioning is a key aspect at the nanometric scale,^9,11^ and chemical synthesis methods do not always succeed at inserting the doping elements inside the nanoparticles core.^12,13^

Alternative bottom-up techniques, where the system undergoes highly non-equilibrium transitions, could be a possible route towards the control of doping in nanoparticles. Since the nineties,^14^ PLAL has proven its reliability and its versatility to synthesise nanomaterials,^15–23^ including doped nanoparticles.^24–30^ PLAL provides clean and ligand-free surfaces,^31^ and can be operated continuously with production rates of several grams per hour.^32,33^ Standard route to obtain doped nanoparticles with this technique is to prepare a doped bulk material as a pellet using solid state reaction before ablation. Preparing a doped pellet is nevertheless not always straightforward since it requires to follow thermodynamic equilibrium pathways. Alternatively, using the solvent as support for the doping agent exhibits high interests because activators can be incorporated within the nanoparticle's matrix in a one step process.

The fundamental mechanisms of PLAL have been studied in a large number of contributions. For each laser pulse, the ablation of the target leads to a hot and dense plasma.^34–37^ In the case of nanosecond pulses, it has been shown that the nascent plasma and the liquid partially merge in the first few hundred nanoseconds.^38^ A fast energy transfer from the laser pulse to the liquid is then supported by the nascent plasma. Solvent molecules are vaporised to form a vapour bubble, mainly composed of the solvent molecules with respect to the ablated matter.^19,39^ Accordingly, atoms from the solvent can significantly contribute to the final stoichiometry of the nanoparticles. Indeed, numerous oxides were obtained starting from pure metal targets ablated in water.^40–47^ Furthermore, carbon nitride nanoparticles were obtained following the ablation of a carbon target in ammonia.^48,49^ Similarly, the ablation of iron targets in various organic solvents led to iron-based nanoparticles including iron carbide (Fe_3_C), iron oxides, amorphous and crystalline iron.^50,51^ The fast cooling (a few microseconds) of the plasma mixed with solvent vaporised molecules leads to the nucleation and growth of nanoparticles with size ranging from few nanometers to a few tens of nanometers. Their composition can combine species from the ablated target and the solvent. Although the role of the solvent molecules was already evidenced, the role of solvated ions or impurities is still poorly documented. Using plasma spectroscopy, Matsumoto *et al.*^52^ showed the transfer of Li^+^ and Na^+^ ions dissolved in the liquid into the plasma. Using SAXS, Letzel *et al.* showed that in the case of a gold target ablation, adding Cl^−^ ions in the aqueous solution allows for a size quenching of the gold nanoparticles. The size quenching is observed early within the cavitation bubble.^53^ Such results demonstrate that the Cl^−^ ions already play an important role during the condensation of the plasma by interacting with the nanoparticle surface during their growth. However, these ions that were added within the solvent could also penetrate the nanoparticles core. While such mechanism has not been investigated so far, it would offer a new route to dope the nanoparticles by ablating undoped materials in a salt solution. In addition, it also raises the question of the penetration of unwanted impurities from the solvent which can hinder the expected properties of the particles.

In order to evaluate the contribution of the solvent as a doping media in PLAL, we performed the ablation of undoped monoclinic phase Gd_2_O_3_ targets immersed in aqueous solutions of EuCl_3_, for various salt concentrations. Gd_2_O_3_:Eu^3+^ is a well-known red luminescent sesquioxyde^26,54,55^ containing gadolinium which is also known as contrast agent for nuclear magnetic resonance imaging,^56,57^ but also as radiosensitizer.^58^ In addition, Eu^3+^ luminescence is sensitive to the crystal structure, since it highly depends on the crystal field symmetry of the crystallographic sites. Eu^3+^ luminescence is thus known as a very efficient structural probe. X-ray diffraction (XRD), selected area electron diffraction (SAED) and high resolution transmission electron microscope (HRTEM) measurements reveal that the generated nanoparticles are in the monoclinic phase. Luminescence properties (emission and excitation) allow to clearly discriminate between emission from Eu^3+^ ions in the core of the monoclinic Gd_2_O_3_ nanoparticles, and emission from the remaining salts or Eu^3+^ adsorbed on the Gd_2_O_3_ surface. Furthermore, the [Eu]/[Gd] ratio has been quantified inside the plasma. Indeed, the amount of vaporised solvent, and then the number of europium atoms inside the plasma are obtained from fast imaging of the laser-induced cavitation bubble, while the number of gadolinium atoms is deduced from product weighing. The deduced [Eu]/[Gd] ratio inside the plasma will be confronted to laser-induced breakdown spectroscopy (LIBS) performed on the dried powder for the highest doped sample.

## 2 Experimental section

### Synthesis (PLAL)

**Fig. 1:**
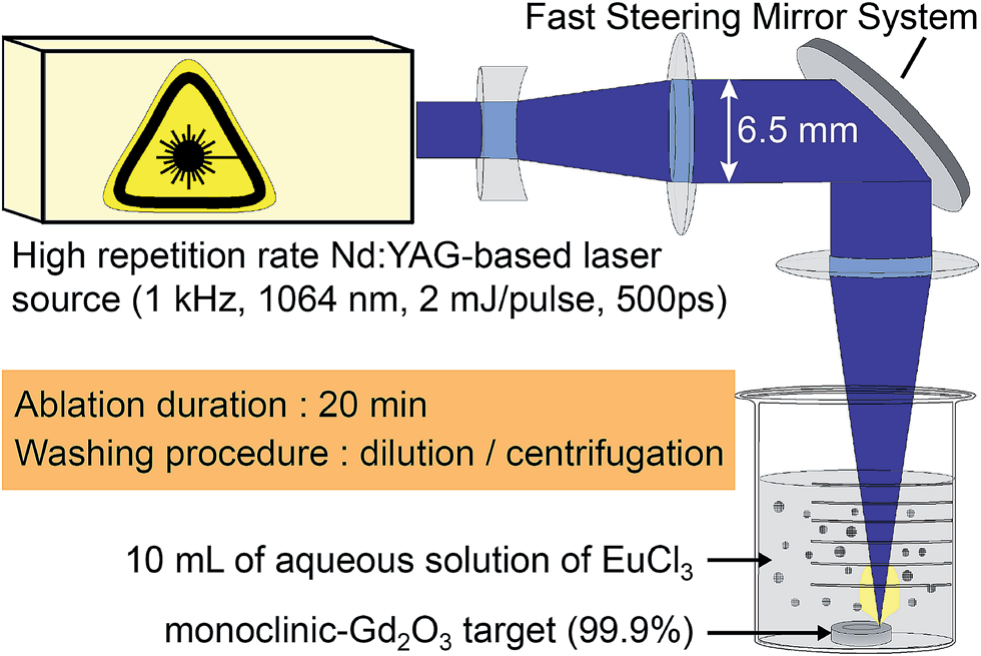
Graphical summary of the synthesis parameters.

A graphical summary of the synthesis parameters is displayed in Fig. 1. The ablation set-up uses a laser source based on a Master Oscillator Power Amplifier (MOPA) architecture from Fibercryst company. The beam from a passively Q-switched kilohertz Nd:YAG laser (1 kHz), operated at 1064 nm, is amplified using a laser gain module (*Taranis* module), which consists in a diode-pumped Nd:YAG single crystal fiber.^59^ Each pulse is 500 ps long and has an energy of 1.5 mJ. The Gaussian TEM00 beam (beam quality *M*^2^ < 1.3) is expended to reach a 1/*e*^2^ diameter 2*w* of 6.5 mm, and is then focused at the surface of the target using a F-Theta scan lens with 160 mm focal length. The expected beam waist 2*w*_0_ on the target is then 44 μm. It leads to a surface power density of 2 × 10^11^ W cm^−2^.

The targets of pure Gd_2_O_3_ are made from a powder of gadolinium(iii) oxide with a purity of 99.9% (Sigma-Aldrich, CAS number 12064-62-9). The powder is pressed and then annealed in an oven at 1400 °C during 6 h. X-ray diffraction shows that the targets are in the monoclinic phase (see Fig. S5 in ESI†).

Synthesis parameters are reported in Table 1. For each synthesis, a target is positioned in a 50 mL beaker filled with 10 mL of a solution of deionized water and Eu(iii) ions. Europium(iii) chloride hexahydrate from Strem Chemicals (CAS number 13759-92-7) with a purity of 99.9% is dissolved in order to reach five different concentrations from 10^−5^ mol L^−1^ to 10^−3^ mol L^−1^. The maximum concentration is defined by a technical limitation of our current protocol. From 5 × 10^−3^ mol L^−1^ the solution remains milky and the ablation efficiency drastically decreases because of the scattering of the laser light. By keeping the salt concentration below 10^−3^ mol L^−1^, we ensure the reproducibility of the ablated mass. Steering mirrors are used to move the ablation spot on the target over a squared area of 11 mm side. The ablation spot is moved of 10 μm every 5 shots forming lines, and each line is 30 μm spaced. The whole solution is gently stirred using a magnetic stirrer to evacuate the produced nanoparticles from the ablation spot. Each synthesis lasts for 20 min (1.2 million shots), then only the supernatant is collected in order to avoid microparticles resulting from a possible target crumbling because of the laser-induced shockwaves.^60^ Nanoparticles are collected by centrifuging the supernatant at 21 036 RCF during 10 min and washed with 10 mL of deionized water to remove europium salt. After centrifugation, the resulting material composed of clean powder is dissolved in 300 μL of deionized water and put into a vessel made from UV plastic (1 cm^2^). For each sample the water is then evaporated in order to obtain uniform films with the same thickness enabling intensity comparison on the measured luminescence spectra.

**Table 1:** Samples preparation for the different characterisation techniques (see text for details). The cut-off diameter of the sub-micronic particles selection is about 800 nm (see Fig. S3 in ESI). The measurement of the ablated mass per pulse is described in ESI. 2.0 ng of nanoparticles are produced for each laser pulse

Characterisation techniques	Ablation duration [min]	Solution volume [mL]	Salt concentration [mol L^−1^]	Purification process
Luminescence, HRTEM, SAED	20	10	10^−5^ to 10^−3^	Sedimentation and 1 washing
Luminescence (sample C)	20	10	None then 10^−3^	Sedimentation and 1 washing
LIBS	20	10	10^−3^	Sedimentation and 10 washings
XRD	400	200	10^−3^	1 washing and sub-micronic particles selection (<800 nm)
Ablated mass per pulse	315 & 380	200	10^−3^	1 washing and sub-micronic particles selection (<800 nm)

A negative control sample (named C) is synthesized in pure deionized water during 20 min. Then EuCl_3_ salt is added to reach a concentration of 10^−3^ mol L^−1^. The colloidal solution is stirred and then washed following the above procedure. The luminescence from the sample C will correspond to adsorbed Eu^3+^ ions on the surface of the nanoparticles.

### Characterisation

#### X-ray diffraction (XRD)

X-ray powder diffraction patterns are recorded at room temperature on a Bruker D8 Advance diffractometer equipped with a sealed Cu X-ray tube and a linear LYNXEYE XE detector. The K_α2_ contribution is removed in the X-ray diffraction pattern shown in Fig. 3a. The XRD are performed on nanoparticles produced with the same protocol (target, laser parameters), but scaled up in order to produce above 50 mg: 400 min long ablation in a 200 mL EuCl_3_ solution at 10^−3^ mol L^−1^. Contrary to the luminescence analysis, XRD is very sensitive to possible residual micronic powder due to laser-induced undoped target crumbling. A purification step is thus added for this analysis. The colloidal solution is poured in 9 cm high tubes (filling height 7 cm) and gently centrifuged at 50 RCF during 10 min. The supernatant is then collected. All the particles larger than 817 nm settle down and are thus removed, and only a few percent of the nanoparticles smaller than 100 nm are removed (see Fig. S3 in ESI†).

**Fig. 2:**
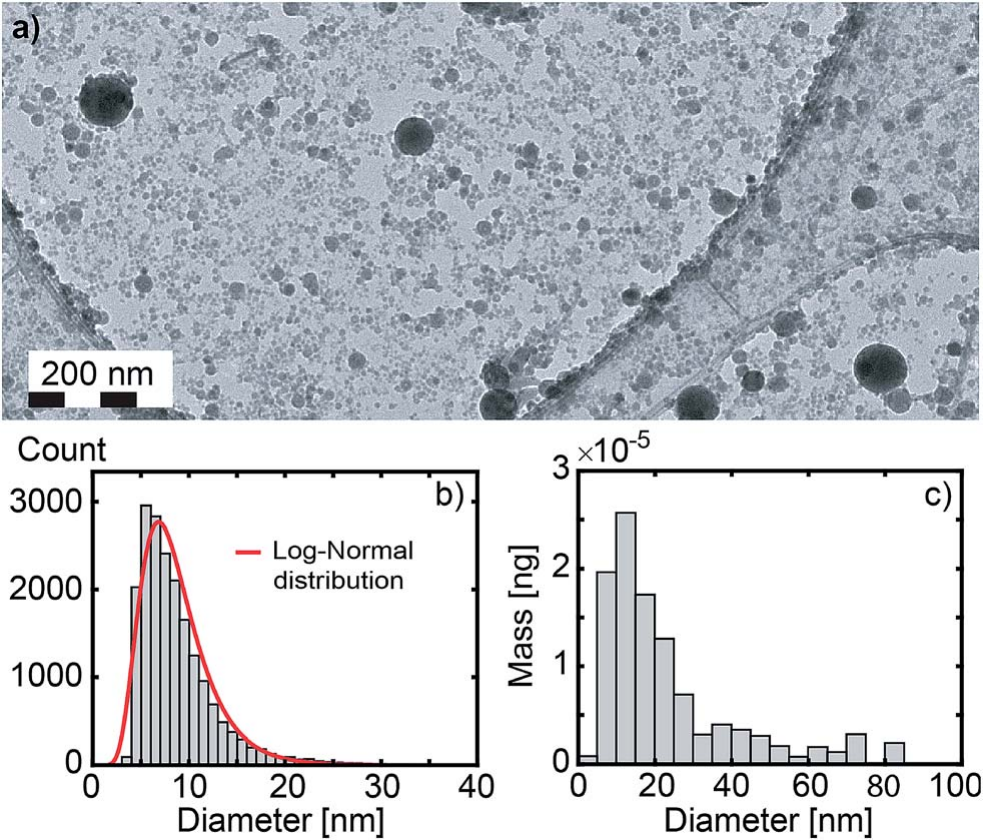
(a) Large field image (×20k) of Gd_2_O_3_ nanoparticles. (b) Size distribution of 19 310 nanoparticles deduced from 3 large field images (see Fig. S4 in ESI†) including image (a). The red curve corresponds to a log-normal distribution fit (median size = 8.0 nm, standard deviation = 3.6 nm). (c) Corresponding mass distribution of the nanoparticles (median size = 19.7 nm).

**Fig. 3:**
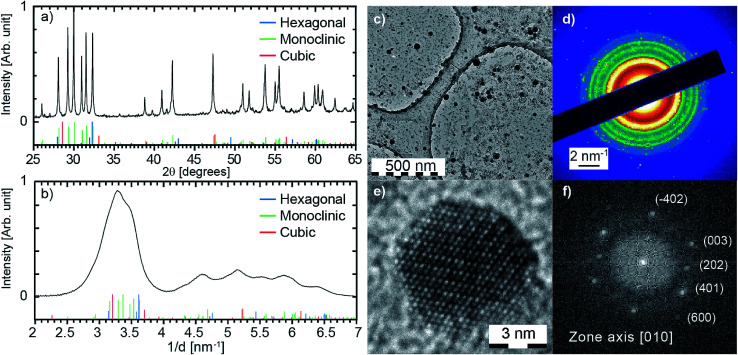
(a) X-ray diffraction pattern of the long synthesis sample (see text for details). The theoretical peak positions for cubic (Powder Diffraction File 00-012-0797 from ICDD), hexagonal (04-016-2410) and monoclinic (00-042-1465) crystal structures are displayed. (b) Diffraction pattern obtained by radial averaging of the selected area electron diffraction (SAED) shown in (d). The selected area corresponds to image (c). (e) HRTEM image of a particle in the monoclinic phase and (f) the associated fast Fourier transform.

#### Transmission electron microscopy (TEM)

For each synthesis, a droplet of the as-produced colloidal solution is poured onto a 400-mesh copper grid covered with ultra-thin carbon on holey carbon support film (reference 01 824 from Ted Pella, Inc.). Transmission Electron Microscopy (TEM) experiments are carried out on a JEOL 2100 HT microscope operating at 200 kV. High resolution images are acquired using a Gatan Orius 200 camera and electronic diffraction patterns are analysed with the Digital Micrograph software from Gatan (see Fig. 3e and f). Selected area electron diffraction (SAED) patterns are averaged radially in order to obtain the diffraction pattern shown in Fig. 3b. Amorphous contribution of the carbon layer was approximated by an exponential and removed. Because a few thousands of particles are probed simultaneously, such a pattern can be interpreted as the powder pattern of the smallest nanoparticles (a few nanometers), even if contribution from larger particles are observed (see bright spots in the SAED from particles of a few tens of nanometers). As a consequence, such a pattern should be more considered as a fingerprint than a quantitative powder pattern. The size distribution in Fig. 2b is obtained from three different large field TEM images, including Fig. 2a. Nanoparticles were automatically detected with the plug-in *Particles Sizer* in *ImageJ*^61^ and diameters were deduced from their areas.

#### Luminescence

**Fig. 4:**
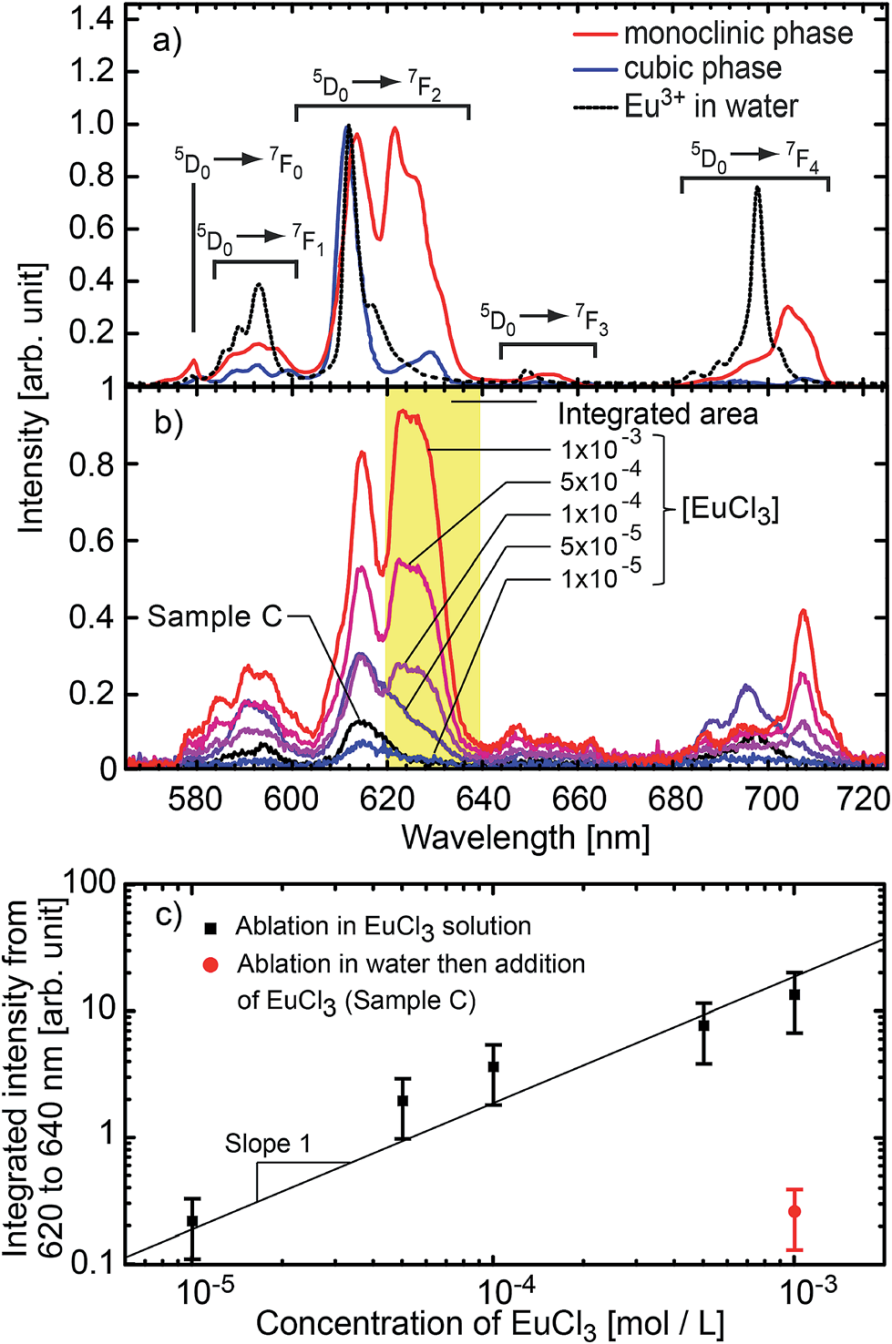
(a) Luminescence spectra of reference Gd_2_O_3_:Eu monoclinic nanospheres,^62^ cubic nanoplatelets,^63^ and europium(iii) chloride hexahydrate salt. (b) Luminescence spectra of the nanoparticles obtained by ablation of pure gadolinia in Eu^3+^ solution at different concentrations. The black line corresponds to the reference sample C, *i.e.* undoped nanoparticles matured in a 10^−3^ mol L^−1^ solution of EuCl_3_ and then washed. The spectral resolution is 2 nm (FWHM). (c) Integrated signal between 620 nm and 640 nm for each concentration (monoclinic signature). The red dot corresponds to the signal from the reference sample C. The error bars correspond to the standard deviation of the amount of ablated material over 5 ablations. Solid line is a reading guide representing a linear power law.

To measure the luminescence spectra displayed in Fig. 4b, samples were excited by a UV LED source from Thorlabs (LED290W) emitting at 293 nm (FWHM = 13 nm). The luminescence was collected by an optical fiber and fed into a monochromator (Triax 320 from Jobin Yvon) with a Peltier-cooled charge coupled device (CCD) array detector. Calibration of the wavelengths was performed with a spectral calibration lamp. Each spectrum displayed corresponds to the accumulation of 15 measurements with an accumulation time of 5 s each. The position of the sample and settings were systematically kept identical in order to achieve the same light collection efficiency and to provide accurate comparison of the intensities.

**Fig. 5:**
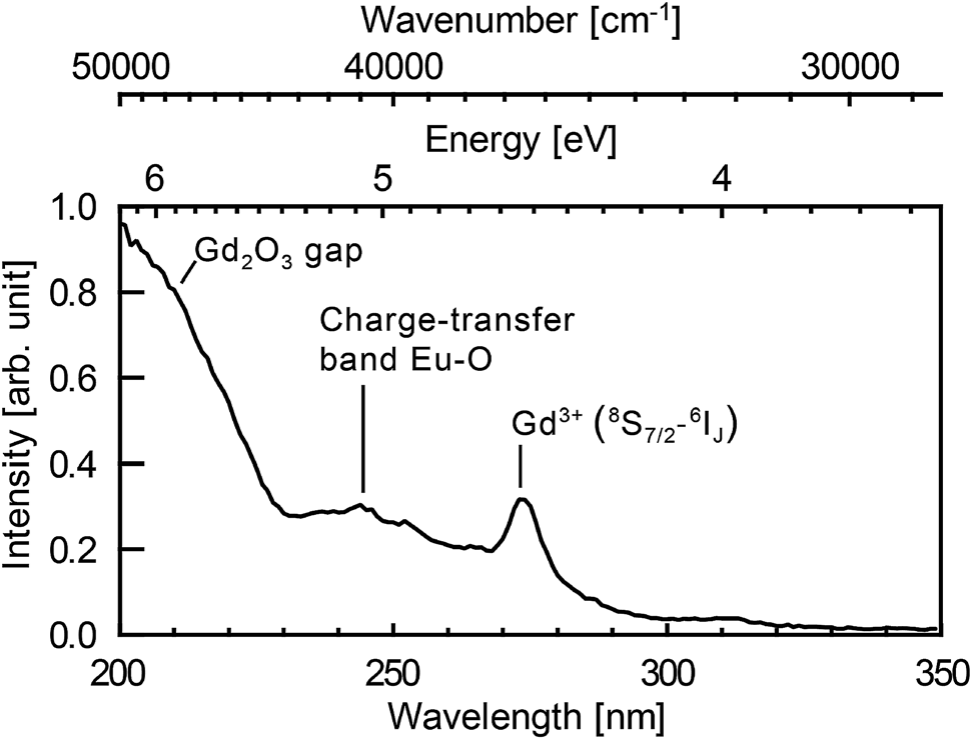
Excitation spectrum of the nanoparticles synthesised in a solution of 10^−3^ mol L^−1^ of europium salt. Emission at 625 nm integrated over 32 nm (FWHM of the spectral resolution). The spectral resolution of the excitation spectrum is 4 nm.

Fig. 5 shows photoluminescence excitation (PLE) spectrum. PLE was measured by a custom made spectrofluorometer 5000M Horiba Jobin Yvon, using a steady state deuterium lamp as excitation source. Single grating monochromators and photon counting photomultiplier based detectors were used for the emission light collection. The spectrum was corrected from the wavelength dependence of the light source intensity.

### [Eu]/[Gd] atomic ratio

Because of the strong interaction between the laser generated plasma and the liquid, a significant amount of liquid and salt is vaporised for each pulse.^39^ The combination of the matter from the vaporised liquid and from the ablated target is the starting material forming the doped nanoparticles. It is thus crucial to evaluate the [Eu]/[Gd] atomic ratio in the plasma, which is deduced from two distinct measurements. On the one hand, the average number of gadolinium atoms in the plasma is obtained from the amount of gadolinium oxide vaporised: 2.0 ng per pulse estimated from product weighing (standard deviation 0.18 ng, see ESI†), leading to 6.6 × 10^12^ Gd atoms per pulse (standard deviation 0.6 × 10^12^). On the other hand, for the average number of europium atoms in the plasma, we first computed the amount of vaporised solvent and then assumed that the europium concentration is the same in the liquid solution and in the vaporised solvent because of the extremely fast vaporisation. Such assumption is justified by recent works from Tamura *et al.* which showed that in the first hundred nanoseconds the nascent plasma and the liquid partially merge.^64^ As a result, the solvent vaporisation is fast enough that the ions dissolved in the liquid can directly transfer into the plasma.^52^ To obtain the amount of vaporised liquid, the dynamics of the bubbles is recorded using shadowgraph.^19,39^

### Shadowgraph imaging

**Fig. 6:**
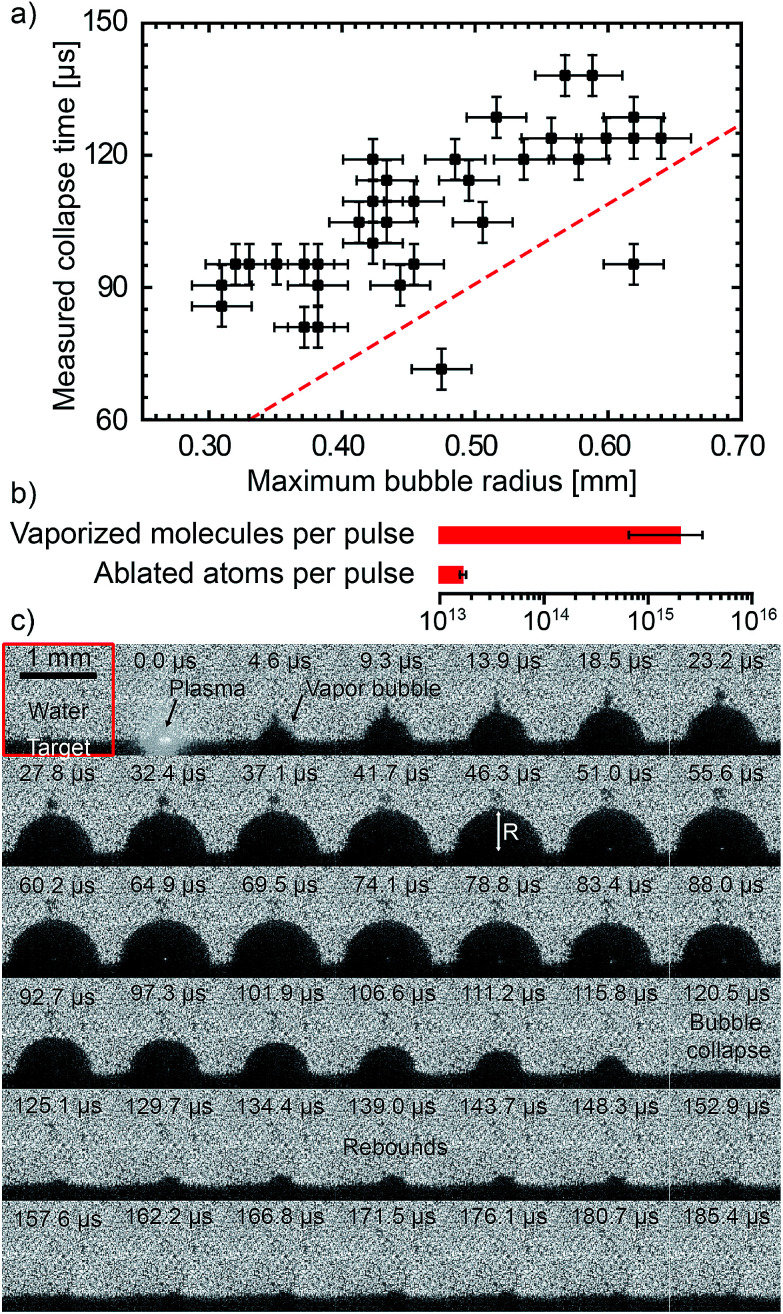
(a) Bubble lifetime (first collapse) as a function of the maximum bubble radius *R*_M_. Dots correspond to 35 individual bubbles. The red dashed line corresponds to the theoretical Rayleigh collapse time^65^ for an oscillation 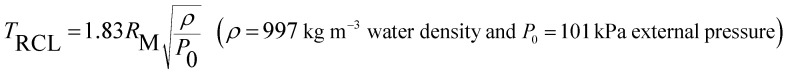. (b) The averaged amount of vaporised molecules per pulse is deduced from the dynamics of 35 bubbles. The error bar corresponds to 68% confidence interval (±standard deviation computed from 35 bubbles). The amount of ablated atoms per pulse is deduced from the product weighing (see ESI†). (c) Shadowgraph snapshots of a laser-generated bubble. The numbers stand for the delay after the nanosecond laser pulse.

The shadowgraph images are collected by an ultrafast camera (Phantom v711 from Vision Research) coupled with a zoom lens system (Zoom 6000 from Navitar). The light source is a continuum HeNe laser (632.8 nm, *P* = 13 mW) coupled to a diffuser. The camera frame rate is 215 800 frames per second. Each image is 128 × 128 px^2^ representing 1.44 × 1.44 mm^2^. The resolution is 22 μm. A complete schematic description of the experimental setup is given in ref. 39. Data from 35 bubbles have been used to provide statistical analysis. The average lifetime of the first oscillation is 106 μs (standard deviation 17 μs). The average maximum bubble radius is 0.46 mm (standard deviation 0.1 mm). The bubble lifetime until the first collapse follows a linear trend with respect to the maximum bubble radius. Such a behaviour is consistent with the theoretical Rayleigh collapse time^65^ (dashed red curve in Fig. 6a), even if the measured lifetimes appeared somewhat longer than the theoretical ones. After the first collapse, rebounds of smaller bubbles can also be observed. The pressure in the vapour bubble is deduced from the Rayleigh–Plesset (RP) equation. The processing of data using RP equation is fully described in our previous work.^39^ The bubbles follow an isentropic process until the first collapse. Assuming the ideal gas law holds, a lower limit of the number of vapour molecules inside the bubble is obtained assuming that the vapour temperature remains lower than the water critical temperature (*T*_c_ = 647 K). The average number 〈*N*〉 of vapour molecules is 2 × 10^15^ (standard deviation 1.3 × 10^15^). The latent heat of vaporisation of 2 × 10^15^ water molecules corresponds to 0.14 mJ (9% of the laser pulse energy). The vapour bubbles are therefore mostly composed of water molecules with respect to the number of ablated atoms (see Fig. 6b). For a 10^−3^ mol L^−1^ EuCl_3_ solution, the deduced amount of europium ions in each bubble is estimated to be 3.6 × 10^10^ (standard deviation 2.4 × 10^10^). It leads to an atomic ratio [Eu]/[Gd] in the plasma of 0.55% (standard deviation 0.37%).

### Laser-induced breakdown spectroscopy (LIBS)

**Fig. 7:**
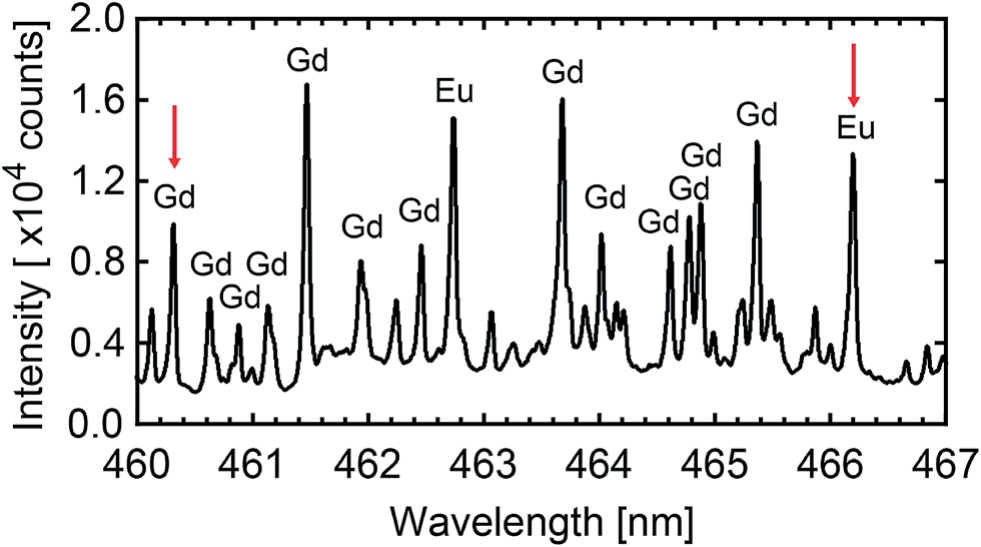
LIBS spectroscopy. Typical atomic emission observed on a sample of Gd_2_O_3_:Eu nanoparticles. The red arrows show both lines used to quantify the atomic ratio [Eu]/[Gd].

The resulting [Eu]/[Gd] atomic ratio in the produced nanoparticles is deduced from LIBS. LIBS method is today considered as a robust analytical method and numerous articles in the literature report on its quantification ability.^66–71^ Moreover, Fig. S1 in ESI† shows that LIBS is reliable to quantified the [Eu]/[Gd] atomic ratio in the case of europium-doped Gd_2_O_3_ for [Eu]/[Gd] extending from 0.1% to 3%. LIBS only consumes a few μg of nanoparticles powder per analysis, which is a significant advantage in regards to other analytical techniques, such as ICP methods which require at least 100 mg of materials. Few hundreds of μg of nanoparticles powder are deposited on a microscope slide with the help of a double-sided adhesive tape. The micro-LIBS instrumentation used Nd:YAG laser pulses of 1064 nm, with an energy of 2 mJ, a pulse duration of 8 ns, and a repetition rate of 20 Hz. Details about the experimental setup can be found in ref. 72 and 73. In order to spatially confine the plasma, the measurements were performed at room temperature with argon gas flowing through the plasma region (1.5 L min^−1^). A beam shutter was used to control the delivery of the laser pulse to the sample. A unique plasma was produced for each sampling position. In total, 30 single shots spectra were recorded for each sample. The light emitted by the plasma plume was collected and focused onto the entrance of an optical fibre bundle. This fibre bundle was composed of 19 fibres with a 200 μm core diameter. It was connected to a Czerny–Turner spectrometer equipped with a 2400 L mm^−1^ grating blazed at 300 nm and an intensified charge-coupled device (ICCD) camera (Shamrock 500 and iStar, Andor Technology). The ICCD camera was synchronised to the Q-switch of the laser and the spectrum acquisition was performed with delay and gate of 2 μs and 2 μs, respectively. For such delayed gate, the plasma cooling leads to a lowest electronic temperature which inhibits the emission from the highest excitation levels, and then the emission of gadolinium (major element). It leads to a better visibility of the selected EuI line. The width of the entrance slit of the spectrometer was set to 15 μm. With this configuration, a typical spectral resolution of 0.05 nm is achieved. All the data treatment was realised by using the lines EuI at 466.18 nm and GdI at 460.29 nm (see Fig. 7). These lines were chosen because they are rather isolated and did not show any apparent saturation effects. The net intensities of both emission lines were extracted using a baseline subtraction. The relative calibration of the [Eu]/[Gd] atomic ratio was performed using a reference sample, containing an [Eu]/[Gd] atomic ratio of 1%.

## 3 Results and discussion

### Nanoparticles characterisation

#### Size distribution

The typical size distribution (particle count) and mass distribution (mass weighted size distribution) are shown in Fig. 2b and c, respectively. The size distribution (particle count) follows a log-normal distribution. Red curve corresponds to the fitted log-normal distribution with a median size of 8.0 nm. Yet, a second population of larger nanoparticles up to 100 nm is observed. Being rare, their number is negligible in comparison to the first population but their mass is not. As shown in Fig. 2c, half of the produced mass corresponds to nanoparticles with a diameter larger than 19.7 nm. Indeed, bimodal size distributions are generally observed,^74–77^ with a first fraction of particles with sizes below 20 nm and a second fraction of particles of a few tens of nanometers. The origin of bimodal size distributions in femto- and picosecond laser ablation in liquids has been addressed by Shih *et al.* using a large-scale atomistic simulation.^78–80^ Looking at the ablation of silver, they suggested a critical role of the dynamic interactions between the ablation plume and the liquid environment. Nucleation and growth of small nanoparticles occur in an expanding metal–liquid mixing region, while the larger particles originate from the fragmentation of the molten layer on the target through Rayleigh–Taylor instabilities.^80^ The bimodal size distribution is expected to appear very early during the plasma cooling. Small angle X-ray scattering (SAXS) supports the early appearance of the bimodal size distribution.^53,81–84^ It also shows that the nanoparticles remain confined in the vapour bubble during a few hundred microseconds, until the bubble collapses. Following this scenario, only the fraction of the smallest particles originated from the plasma condensation is expected to be doped in the core.

#### Crystallographic phase

First, XRD measurement performed on the obtained nanoparticles at a large scale is shown in Fig. 3a. The pattern clearly corresponds to a monoclinic phase, and no obvious impurity phases are detected. The Scherrer equation is applied to peak widths (FWHM) of the six main peaks observed in the powder diffraction pattern between 28.15° and 32.4° ((111), (401), (−402), (003), (−310) and (−112)). It leads to an average domain size of 54 nm. It appears that the diffraction pattern is mostly governed by the largest particles, which is consistent with a median size of 19.7 nm for the mass weighted size distribution (see Fig. 2c). Therefore, SAED and HRTEM are also employed to investigate the crystal structure of the smallest nanoparticles (<10 nm). Typical low resolution TEM and high resolution TEM captured on the sample synthesised with 10^−4^ mol L^−1^ of EuCl_3_ salt are displayed in Fig. 3c and e respectively. The diffraction pattern is deduced from a radial averaging of the SAED acquired on a large area [see Fig. 3b–d]. Such a pattern being acquired over few thousand nanoparticles is similar to their powder diffraction pattern. Again, the monoclinic Gd_2_O_3_ diffraction pattern explains very well the experimental observation. Then FFT has been performed on about 25 high resolution images of particles as shown in Fig. 3e and f. Crystallographic planes always match monoclinic phase. Altogether, XRD, SAED and HRTEM show that a monoclinic phase is obtained for both the largest and the smallest gadolinium oxide nanoparticles. This is consistent with previous reports on PLAL-generated doped and undoped-Gd_2_O_3_ nanoparticles of above 10 nm. Indeed, monoclinic Gd_2_O_3_ nanoparticles have been produced from laser ablation of a pure Gd target in water.^41^ Monoclinic nanoparticles of co-doped Gd_2_O_3_ phosphors have been produced from laser ablation of co-doped Gd_2_O_3_ target, namely Gd_2_O_3_:Er,Yb^27^ and Gd_2_O_3_:Yb,Tm.^30^

At room pressure and temperature, the cubic phase is the most stable form for Gd_2_O_3_ bulk crystal. Monoclinic phase is observed for temperature higher than 1130 °C.^85^ However, a crossover in polymorph stability is often reported at the nanoscale. For several oxides such as Al_2_O_3_, TiO_2_, and ZrO_2_, the differences in surface energy stabilise polymorphs that are metastable in the bulk when a critical surface area is exceeded.^86^ Same behaviour has been reported for nanoparticles synthesised by laser ablation of a Gd_2_O_3_:Eu pellet in a Low Energy Cluster Beam Deposition setup. The particles with a size lower than 2.8 nm are mainly in the monoclinic phase while the larger ones are mainly in the cubic phase.^54^ PLAL seems to enable the stabilisation of larger particles in the monoclinic phase. Large energy deposition, leading to a plasma of a few thousands of kelvin,^19,37^ combined with very fast cooling rate above 10 K ns^−1^ (ref. 19) can contribute to the stabilisation of metastable phases.

### Evidence of core doping: luminescence properties

The Eu^3+^ emission strongly depends on the crystal field and thus on the symmetry of the crystallographic sites. The radiative transition intensities can be deduced from Judd–Ofelt theory,^87,88^ and extensive simulations have been performed to look at the influence of the crystal-field on the trivalent lanthanide emission and absorption.^89–93^ As an example, in cubic-Gd_2_O_3_, Eu^3+^ are in crystal sites with *C*_2_ symmetry and *S*_6_ symmetry.^47,94^ It leads to an emission spectrum which can be easily distinguished from the emission spectrum of Eu^3+^ in monoclinic-Gd_2_O_3_ characterized by three non-equivalent *C*_s_ crystallographic sites^47^ (see Fig. 4a). The luminescence lines are thus a signature of the occupied crystallographic sites. Moreover, the crystal field on surface sites is different from the crystal field in the core of the matrix.

Fig. 4b shows the emission spectra of the samples when excited at 293 nm (FWHM 13 nm). This excitation wavelength covers both the charge-transfer band Eu–O and the ^8^S_7/2_ → ^6^I_*J*_ intra-configurational transition of Gd^3+^ (see Fig. 5). It then favours the excitation of the europium ions in the vicinity of gadolinium or oxygen atoms. Eu_2_O_3_ is not expected since a strong luminescence quenching appears in pure Eu_2_O_3_. In Fig. 4b, we can observe the europium emission corresponding to the intra-configurational transitions ^5^D_0_ → ^7^F_*J*_ for *J* in [0, 4] (see Fig. 4a). For samples ablated in EuCl_3_ solutions (coloured curves), luminescence spectra have features consistent with Eu^3+^ emission for europium in the *C*_s_ crystallographic sites of monoclinic Gd_2_O_3_ (620–640 nm band and 705–720 nm band). The observed emission broadening comes from an inhomogeneous broadening commonly reported for nanoparticles of lanthanide-doped sesquioxydes when they are not annealed.^24,26,55,95,96^ Moreover, Gd^3+^ and Eu^3+^ have identical outer electronic orbitals and Gd_2_O_3_ is an ionic structure, it is then highly unlikely to find Eu^3+^ in an interstitial position. At last, a more direct assessment of the core doping comes from the negative control sample (sample C). It corresponds to pure Gd_2_O_3_ nanoparticles matured in a solution of EuCl_3_ (10^−3^ mol L^−1^). Sample C only exhibits moderate emission in the 580–600 nm, 610–620 nm and 680–705 nm wavelength ranges which corresponds to the emission from surface-adsorbed Eu^3+^ or remaining europium salt, but not from Eu-doped monoclinic Gd_2_O_3_ (see Fig. 4a). Emission and excitation spectra, as well as the negative control sample, show that the europium ions penetrate the core of the nanoparticles during the growth.

### Doping efficiency

In the following, we will quantify the amount of europium doping atoms inside Gd_2_O_3_ matrix. A first insight is given by the luminescence intensity comparison. Fig. 4c shows the integrated signal between 620 and 640 nm which corresponds to a characteristic feature of Eu^3+^ emission in monoclinic Gd_2_O_3_. Even if complex growth mechanisms involving highly non-equilibrium processes are at stake, our results demonstrate that the luminescence intensity is proportional to the EuCl_3_ concentration over two orders of magnitude. The linear trend also shows that the luminescence concentration quenching mechanism does not appear, which is confirmed by the emission lifetime remaining milliseconds (see ESI†). The absence of quenching shows that the atomic ratio [Eu]/[Gd] remains below a few percents in the nanoparticles.^97,98^ In addition, the ratio is measured using LIBS on powder synthesised in a solution of 10^−3^ mol L^−1^, then washed ten times to avoid any remaining salt (see Fig. S2 in ESI†). LIBS measurement leads to an atomic ratio [Eu]/[Gd] equal to 0.83% (standard deviation 0.015%). This value is reliable with respect to the LIBS sensitivity (see Fig. S1 in ESI†). Moreover, this value is consistent with the ratio in the plasma equal to 0.55% (standard deviation 0.37%).

### Mechanism of nanoparticles generation

The nanoparticles with a size bellow a few tens of nanometers are assumed to nucleate and grow during the fast plasma cooling.^78–80^ In such a scenario, the ratio [Eu]/[Gd] in the nanoparticles core would linearly depend on the ratio [Eu]/[Gd] in the plasma. Indeed, Gd and Eu atoms are very similar in size and electronic properties and almost no differences are to be expected in the condensation process. Our findings are consistent with such a scenario, including a fast vaporisation of the solvent during the plasma lifetime,^52^ and nucleation/growth mechanisms taking places during the fast plasma cooling. Moreover, the fast cooling rate above 10 K ns^−1^ (ref. 19) can contribute to the stabilisation of the monoclinic crystal structure which is supposed to be metastable for the particle size reported in our work.

## 4 Conclusions

Laser ablation of a gadolinium oxide in an europium chloride solution leads to europium doped gadolinium oxide nanoparticles in the monoclinic structure. We then demonstrated experimentally that impurities from the solvent can effectively penetrate the core of the nanoparticles as they grow. With our technique, the doped nanomaterials are indeed obtained in a highly versatile single step process. We reached a doping of about 1% ([Eu]/[Gd] atomic ratio), and we showed that the doping concentration is proportional to the initial concentration of europium ions in the solvent over two orders of magnitude. This finding is an additional confirmation to the current understanding of the generation process of the nanoparticles of a few nanometers in PLAL: (i) the interaction between the laser generated plasma and the liquid leads to solvent vaporisation, and (ii) following a fast cooling of the plasma, nucleation and growth of the nanoparticles occurs in an environment composed of ablated target and vaporised solvent. This work could open new ways for the synthesis of doped nanomaterials, but it also raises the question of undesired penetration of impurities in laser-generated nanoparticles in liquids.

## Conflicts of interest

There are no conflicts to declare.

## Supplementary Material

NA-001-C9NA00223E-s001
