# Supplementary material for: Doping nanoparticles using pulsed laser ablation in a liquid containing the doping agent
Source: Nanoscale Adv. 2019 Aug 30;1(10):3963–72. doi: 10.1039/c9na00223e (PMC9419851; doi:10.1039/c9na00223e)
Supplement: NA-001-C9NA00223E-s001 [file NA-001-C9NA00223E-s001.pdf]

Electronic Supplementary Material (ESI) for Nanoscale Advances.
This journal is © The Royal Society of Chemistry 2019

Doping nanoparticles using pulsed laser ablation in a liquid containing the doping agent - Supplementary material

Arsène Chemin, * Julien Lam, † Gaetan Laurens, * Florian Trichard, *
Vincent Motto-Ros, * Gilles Ledoux, * Vítězslav Jarý, ‡ Valentyn Laguta, †
Martin Nikl, † Christophe Dujardin, * David Amans, §*

Quantitative sensitivity of LIBS

Figure 1 shows an assay performed on the quantitative sensitivity of LIBS in the case of europium-doped Gd_2O_3. The experimental conditions detailed below are identical to those reported in the manuscript with the exception of the Gd I emission at 462.44 nm was used, which is similar to the Gd I at 460.29 nm (both are rather isolated and did not show any apparent saturation effects). For europium the lines Eu I at 466.18 nm is used (see Figure 1a). The micro-LIBS instrumentation used Nd:YAG laser pulses of 1064 nm, with an energy of 2 mJ, a pulse duration of 8 ns, and a repetition rate of 20 Hz. Details about the experimental setup can be found in references [1, 2]. In order to spatially confine the plasma, the measurements were performed at room temperature with argon gas flowing through the plasma region (1.5 $L.min^{-1}$). A beam shutter was used to control the delivery of the laser pulse to the sample. A unique plasma was produced for each sampling position. In total, 30 single shots spectra were recorded for each sample. The light emitted by the plasma plume was collected and focused onto the entrance of an optical fibre bundle. This fibre bundle was composed of 19 fibres with a 200 μm core diameter. It was connected to a Czerny−Turner spectrometer equipped with a 2400 l/mm grating blazed at 300 nm and an intensified charge−coupled device (ICCD) camera (Shamrock 500 and iStar, Andor Technology). The ICCD camera was synchronised to the Q-switch of the laser and the spectrum acquisition was performed with delay and gate of 2 μs and 2 μs, respectively. For such delayed gate, the plasma cooling leads to a lowest electronic temperature which inhibits the emission from the highest excitation levels, and then the emission of gadolinium (major element). It leads to a better visibility of the selected Eu I line. The width of the entrance slit of the spectrometer was set to 15 μm. With this configuration, a typical spectral resolution of 0.05 nm is achieved. The net intensities of both emission lines were extracted using a baseline subtraction.

Four reference samples with an [Eu]/[Gd] atomic ratio extending from 0.1 % to 3 % are measured (Particles synthesized by polyol-mediated synthesis [3]). Figure 1b shows measured [Eu]/[Gd] atomic ratio performed on the four reference samples. The linear regression conducted to a good linearity (R^2=0.996) and confidence hyperboles [4] were plotted (95% confidence level). It shows that the concentration measured for the 10^{-3} mol/L sample lies in the concentration range for which LIBS measurements are relevant.

*Univ Lyon, Univ Claude Bernard Lyon 1, CNRS, Institut Lumière Matière, F-69622, Villeurbanne, France.
†Center for Nonlinear Phenomena and Complex Systems, Code Postal 231, Université Libre de Bruxelles, Boulevard du Triomphe, 1050 Brussels, Belgium
‡Inst Phys AS CR, Cukrovarnicka 10, Prague 16200, Czech Republic
§corresponding author

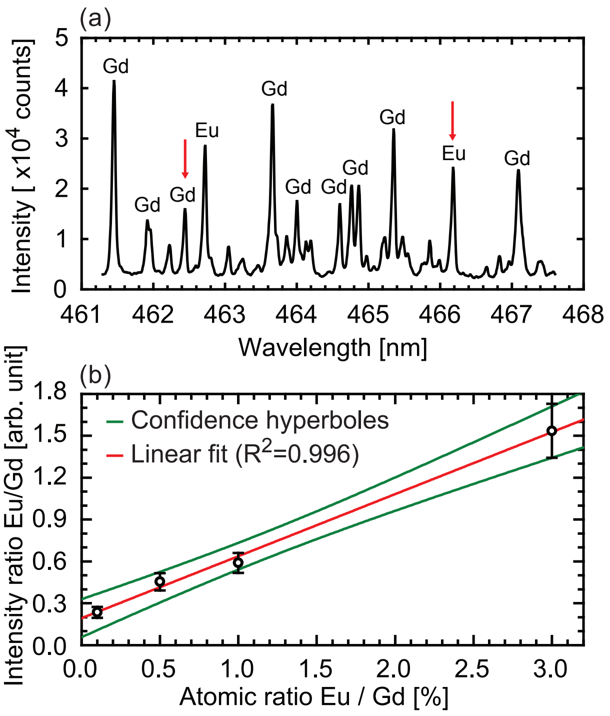

Figure 1: Measurement on samples washed only one times. (a) LIBS spectroscopy. Typical atomic emission observed on a sample of Gd_2O_3:Eu^{3+} nanoparticles. The red arrows show both lines used to quantify the atomic ratio [Eu]/[Gd] in (b). (b) Four reference samples (dots) from polyol-mediated synthesis [3], with a [Eu]/[Gd] atomic ratio range extending from 0.1% to 3%. The error bars correspond to the standard deviation computed from 30 successive measurements. The linear regression conducted to a rather good linearity (R^2=0.996). Confidence hyperboles are plotted in green (95% confidence level).

Purification procedure efficiency evaluated by LIBS

Because each characterisation requires different quality of purification and amount of matter, three synthesis were achieved in the same conditions than described in the main manuscript for an $EuCl_3$ salt concentration of 10^{-3} mol.L^{-1}. The first one (1P, for Luminescence) was washed according to the previous protocol. The second one (2P, for DRX) was washed twice and the third one (10P, for LIBS) was washed ten times. Figure 2a shows the normalised luminescence spectra of the samples recorded on a FS5 Spectrofluorometer (excitation from 265 nm to 275 nm, spectral resolution 1 nm, integration time 0.3 s, each spectrum is averaged over five acquisitions). Figure 2b shows the amount of Europium in each sample evaluated by LIBS. We can assume than after ten washing, there is no remaining solvated salt. It also appears that the purification has no significant influence on the luminescence spectra. This is consistent with the excitation wavelengths used, which cover both the charge-transfer band Eu-O and the $^8S_{7/2} \rightarrow {}^6I_J$ intra-configurational transition of Gd^{3+}. It then favours the excitation of the europium ions in the core of the Gd_2O_3 matrix, or surface-adsorbed europium.

Ablated mass per pulse

The amount of ablated mass is determined by weighting size selected particles resulting from two different ablations. They are respectively performed during 380 min and 315 min in 200 mL of $EuCl_3$ solution at 10^{-3} mol.L^{-1}. The ablation duration enables to produce a significant amount of material. After ablation, the colloidal solution is poured in 9 cm high tubes (filling height 7 cm) and gently centrifuged at 50 RCF (rotor diameter 10.4 cm) during 10 min. The supernatant is then collected. All the particles larger than 817 nm settle down and are thus removed, and only a few percents of the nanoparticles smaller than 100 nm are removed (see Figure 3). The supernatant is centrifuged at 13130 RCF during 14 h in order to precipitate the nanoparticles. The targets, the particles larger than 817 nm and the nanoparticles (<817 nm) are dried out in an oven at 90 ^{o}C during 10 h and weighted on a micro-balance separately. 2.0 ng (Standard deviation 0.18 ng) of nanoparticles is produced for each laser pulse, which corresponds to only 13 % of the ablated material. According to the mass weighted size distribution, it corresponds to nanoparticles smaller than 100 nm.

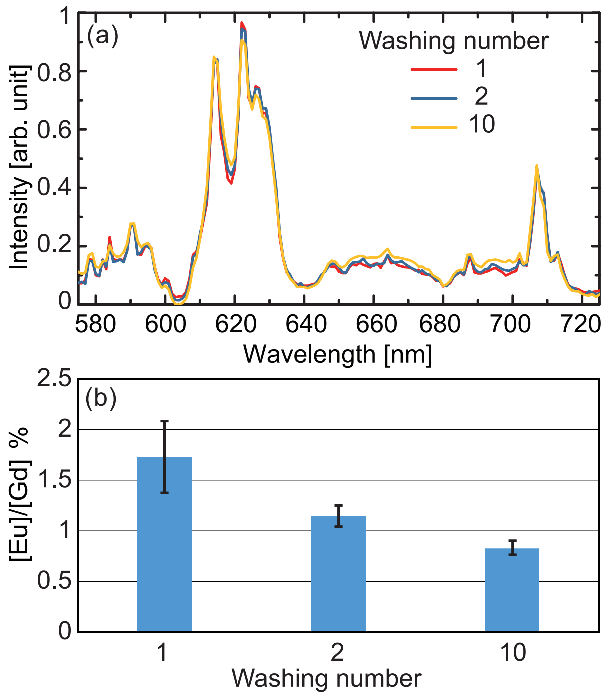

Figure 2: a) Luminescence spectra of the nanoparticles obtained by ablation of pure gadolinia in Eu^{3+} solution at 10^{-3} mol.L^{-1} washed 1, 2 and 10 times. b) [Eu]/[Gd] percentage in the corresponding samples measured by LIBS. Error bars correspond to the confidence interval of 99.7% (three times the standard deviation).

Size distribution

The typical size and mass distribution are calculated from three different large field TEM images shown in figures 4a,b,c. Nanoparticles were automatically detected using *ImageJ* software and its plug-in *Particles Sizer* [5]. Figure 4d shows the detected particles in red from the image c. The diameters were defined as the area equivalent diameter.

X-ray diffraction

The X-ray diffraction patterns displayed in figure 5 clearly show that both nanoparticles and targets are monoclinic Gd_2O_3. No obvious impurity phases are detected. The intensities in the nanoparticle's pattern fit very well the theoretical powder diffraction intensities. The peaks are broadened compared to the target's pattern. According to Scherrer equation applied to peak widths, the diffraction pattern is governed by the larger particles. The intensities in the target's pattern deviate from the theoretical powder diffraction intensities. It shows that the elaboration procedure of the pellets (annealed pressed powder) leads to a partial grain orientation.

Excitation spectrum and luminescence decay

Figure 6 shows photoluminescence excitation (PLE) spectrum and photoluminescence decay curve. They were measured by a custom made spectrofluorometer 5000M Horiba Jobin Yvon, using a steady state deuterium lamp (PLE spectrum) and a microsecond xenon pulsed flash lamp (PL decay) as excitation sources. Single grating monochromators and photon counting photomultiplier based detectors were used for the emission light collection. Spectra were corrected for the instrumental response and a convolution procedure was applied to the decay curves to determine true decay times (SpectraSolve software package, Ames Photonics).

The luminescence lifetime in the millisecond range (see figure 6b) is consistent with the emission from

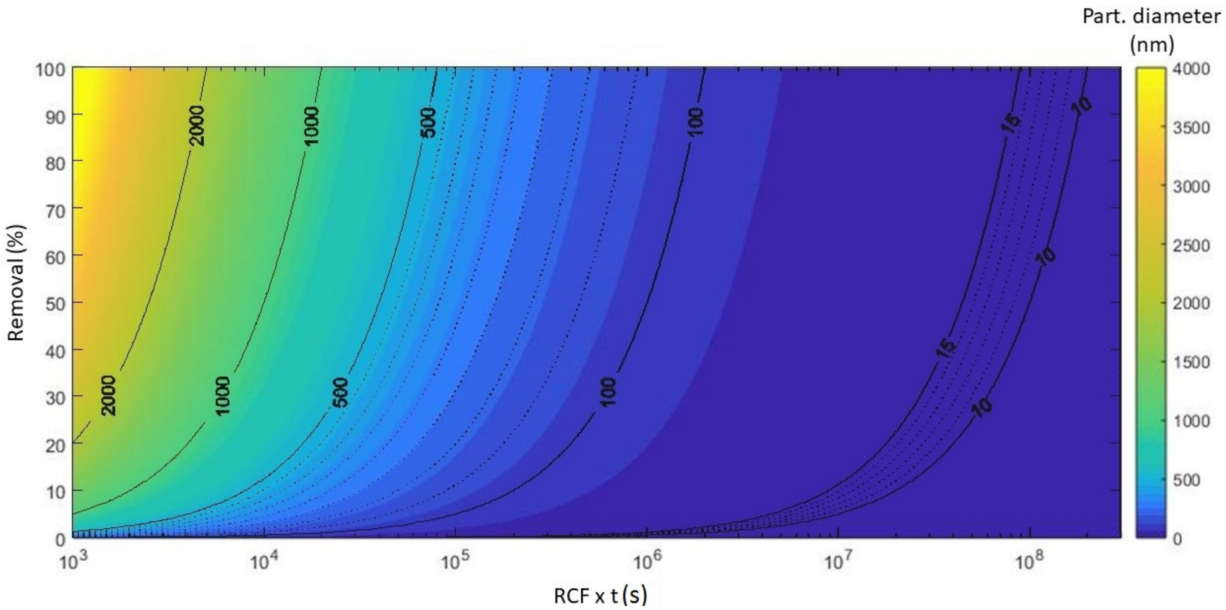

Figure 3: Percentage of removal for different size of Gd_2O_3 particles (density 7.41 g.cm^{-3}) in water as a function of the Relative Centrifugal Force times the centrifugation duration. The tube filling height is 7 cm. The velocity of sedimentation is based on the Stoke's law. The sub-micronic particles selection performed corresponds to $RCF \times t = 50 * 600 = 3 \times 10^4$.

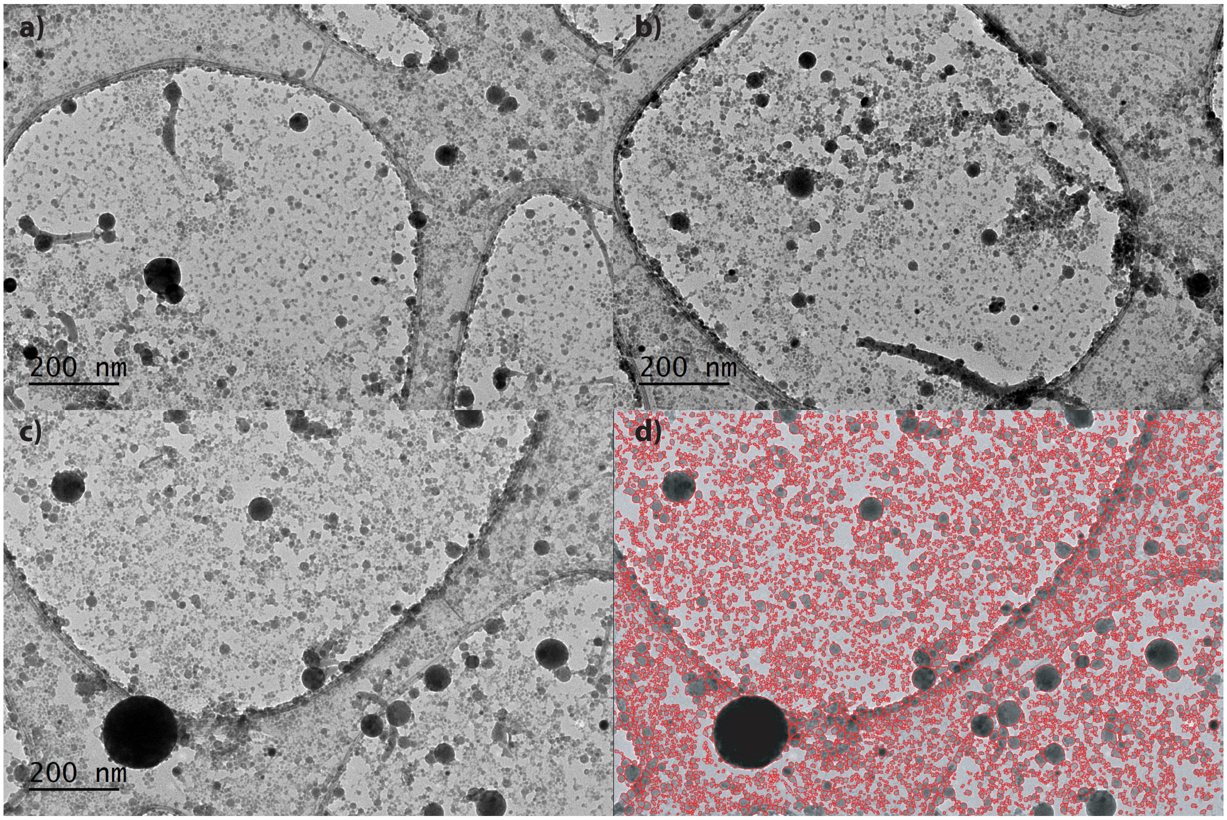

Figure 4: a,b,c) Large field images used to compute the size distribution. d) corresponds to the image processing performs on image c) using the plug-in *Particles Sizer* [5] of *ImageJ* software.

forbidden f-f transition. The biexponential fitting is consistent with the reported in-homogeneous broadening of the luminescent spectra.

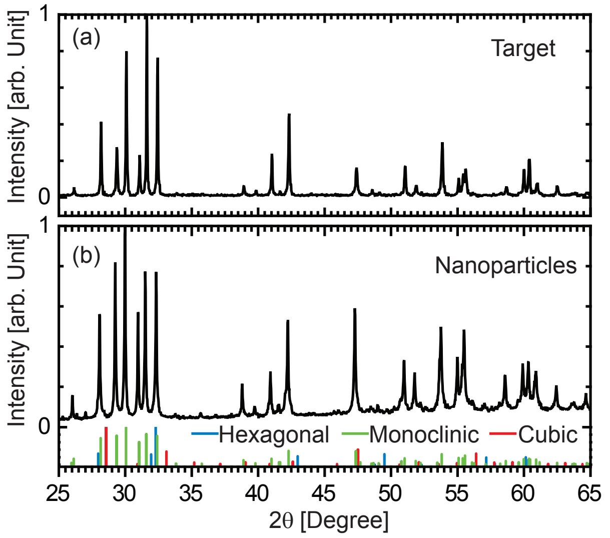

Figure 5: X-ray diffraction pattern measured on a bulk target (a), and on the dry nanoparticles (b). The theoretical peak positions for cubic (Powder Diffraction File 00-012-0797 from ICDD), hexagonal (04-016-2410) and monoclinic (00-042-1465) crystal structures are displayed.

Reference

[1] V. Motto-Ros, E. Negre, F. Pelascini, G. Panczer, and J. Yu. Precise alignment of the collection fiber assisted by real-time plasma imaging in laser-induced breakdown spectroscopy. Spectroc. Acta Pt. B, 92:60–69, FEB 1 2014.

[2] L. Sancey, V. Motto-Ros, B. Busser, S. Kotb, J. M. Benoit, A. Piednoir, F. Lux, O. Tillement, G. Panczer, and J. Yu. Laser spectrometry for multi-elemental imaging of biological tissues. Sci. Rep., 4:6065, AUG 14 2014.

[3] M. A. Flores-Gonzalez, C. Louis, R. Bazzi, G. Ledoux, K. Lebbou, S. Roux, P. Perriat, and O. Tillement. Elaboration of nanostructured Eu^{3+}-doped Gd_2O_3 phosphor fine spherical powders using polyol-mediated synthesis. Appl. Phys. A, 81(7):1385–1391, NOV 2005.

[4] Jean-Michel Mermet. Calibration in atomic spectrometry: A tutorial review dealing with quality criteria, weighting procedures and possible curvatures. Spectroc. Acta Pt. B, 65(7):509–523, JUL 2010.

[5] Thorsten Wagner and Jan Eglinger. thorstenwagner/ij-particlesizer: v1.0.9 Snapshot release, jun 2017.

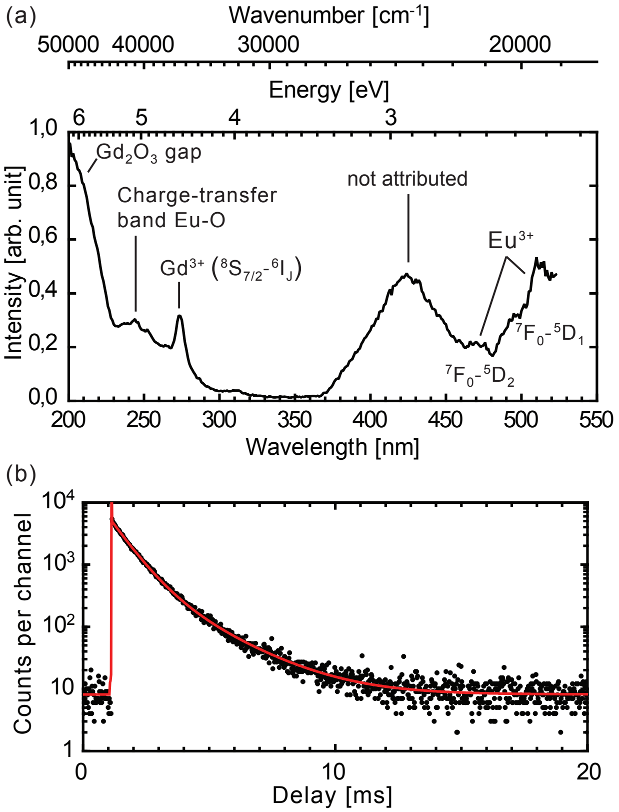

Figure 6: (a) Excitation spectrum of the nanoparticles synthesized in a solution of 10^{-3} mol.L^{-1} of europium salts. Emission at 625 nm integrated over 32 nm (FWHM of the spectral resolution). The spectral resolution of the excitation spectrum is 4 nm. (b) The black curve corresponds to the luminescence decay for excitation at 274 nm (^6I_J level of Gd^{3+}) and emission at 630 nm. The spectral resolution is 6 nm on both emission and excitation. The red curve corresponds to a biexponential fitting : 4382 exp(-t/725.3 μs) + 667 exp(-t/1977 μs)+8.
